# Continuous monitoring of intrinsic PEEP based on expired CO_2_ kinetics: an experimental validation study

**DOI:** 10.1186/s13054-019-2430-9

**Published:** 2019-05-29

**Authors:** Sarah Heili-Frades, Fernando Suarez-Sipmann, Arnoldo Santos, Maria Pilar Carballosa, Alba Naya-Prieto, Carlos Castilla-Reparaz, Maria Jesús Rodriguez-Nieto, Nicolás González-Mangado, German Peces -Barba

**Affiliations:** 1 Intermediate Respiratory Care Unit, Pulmonology Department, IIS-Fundación Jiménez Díaz, UAM, CIBERES, Madrid, Spain; 2 Servicio de Medicina Intensiva, Hospital Universitario de la Princesa, Madrid, Spain; 3 CIBER de Enfermedades Respiratorias, Instituto Carlos III, Madrid, Spain; 4 Department of surgical Sciences, Section of Anesthesia and Critical Care, Hedenstierna Laboratory, Uppsala University Hospital, Uppsala, Sweden; 5 ITC Ingeniería y Técnicas Clínicas, CIBER de Enfermedades Respiratorias (CIBERES), Madrid, Spain; 6 Department of Experimental Surgery, IIS-Fundación Jiménez Díaz, Madrid, Spain

**Keywords:** Intrinsic PEEP, Dynamic hyperinflation, CO_2_, Volumetric capnography, Mechanical ventilation

## Abstract

**Background:**

Quantification of intrinsic PEEP (PEEPi) has important implications for patients subjected to invasive mechanical ventilation. A new non-invasive breath-by-breath method (etCO_2_D) for determination of PEEPi is evaluated.

**Methods:**

In 12 mechanically ventilated pigs, dynamic hyperinflation was induced by interposing a resistance in the endotracheal tube. Airway pressure, flow, and exhaled CO_2_ were measured at the airway opening. Combining different I:E ratios, respiratory rates, and tidal volumes, 52 different levels of PEEPi (range 1.8–11.7 cmH_2_O; mean 8.45 ± 0.32 cmH_2_O) were studied. The etCO_2_D is based on the detection of the end-tidal dilution of the capnogram. This is measured at the airway opening by means of a CO_2_ sensor in which a 2-mm leak is added to the sensing chamber. This allows to detect a capnogram dilution with fresh air when the pressure coming from the ventilator exceeds the PEEPi. This method was compared with the occlusion method.

**Results:**

The etCO_2_D method detected PEEPi step changes of 0.2 cmH_2_O. Reference and etCO_2_D PEEPi presented a good correlation (*R*^2^ 0.80, *P* < 0.0001) and good agreement, bias − 0.26, and limits of agreement ± 1.96 SD (2.23, − 2.74) (*P* < 0.0001).

**Conclusions:**

The etCO_2_D method is a promising accurate simple way of continuously measure and monitor PEEPi. Its clinical validity needs, however, to be confirmed in clinical studies and in conditions with heterogeneous lung diseases.

**Electronic supplementary material:**

The online version of this article (10.1186/s13054-019-2430-9) contains supplementary material, which is available to authorized users.

## Introduction

Intrinsic PEEP results from a delayed lung emptying when expiration is interrupted by the next inspiratory effort, before the lung has reached a static equilibrium volume. Its magnitude is affected by tidal volume (VT), expiratory time, airway resistance, and respiratory system compliance [1–3] and has important clinical consequences in a wide range of respiratory conditions, being particularly relevant during mechanical ventilation (MV).

During spontaneously breathing, PEEPi can only be determined by simultaneously recording esophageal pressure and airway flow tracings. Under passive invasive MV, the simple observation of real-time airflow and airway pressure vs time waveforms at the point of end-expiration can easily identify the presence of PEEPi [4–6]. In fact, the persistence of airflow at end-expiration is an indication that alveolar pressure remains higher than the applied external PEEP [7]. This level can then be quantified by performing an expiratory hold maneuver, the so-called occlusion method (OM), allowing this pressure to reach the measurement site in the ventilator. Such a maneuver is not suitable during spontaneous breathing and provides only intermittent measurements.

We have developed a new method for determining and quantifying the level of PEEPi level based on the end-tidal dilution of the capnogram curve (etCO_2_D) while constant pressure is applied in the airway during expiration. This design allows an assessment of the individual level of PEEPi on a breath-by-breath basis. In the current study, we evaluated the performance of this new method comparing it with the occlusion method in an experimental porcine model.

## Methods

### Animal instrumentation and experimental setup

This study was approved by the animal ethics committee of the institution. After intramuscular injection of atropine (1 mg kg^−1^), xylazine (10 mg kg^−1^), and ketamine (10 mg kg^−1^), animals were intubated and mechanically ventilated through a 7-mm endotracheal tube. Anesthesia and analgesia were maintained by infusion of propofol (5–10 mg kg^−1^ min^−1^) and remifentanil (0.2–0.6 mcg kg^−1^ min^−1^). A venous catheter was inserted into the main auricular vein for drug and fluid administration and a femoral artery by sterile surgical preparation for hemodynamic monitoring. Ringer’s lactate solution was continuously infused at a rate of 5 mL kg^−1^ h^−1^; heart rate, surface ECG, and oxygen saturation were continuously monitored (SC9000, Siemens, Erlangen, Germany). During animal instrumentation and “washout” periods, a Servo I ventilator (Servo-i, Maquet, Critical Care, Solna, Sweden) was used, while during the experimental setup, a single-limb BiPAP respirator (Respironics BiPAP ST) was used.

For volumetric capnography (Vcap) measurements, a mainstream CO_2_ sensor mounted on an adaptor including a fixed orifice pneumotachograph was placed between the tracheal tube and the Y-piece of the ventilator tubing and connected to a NICO monitor (Philips/Respironics, Wallingford, CT, USA). Vcap data and lung mechanics were continuously recorded by a specific software (DataCol, Philips/Respironics, Wallingford, CT, USA).

### The end-tidal CO_2_ dilution method

**Fig. 1:**
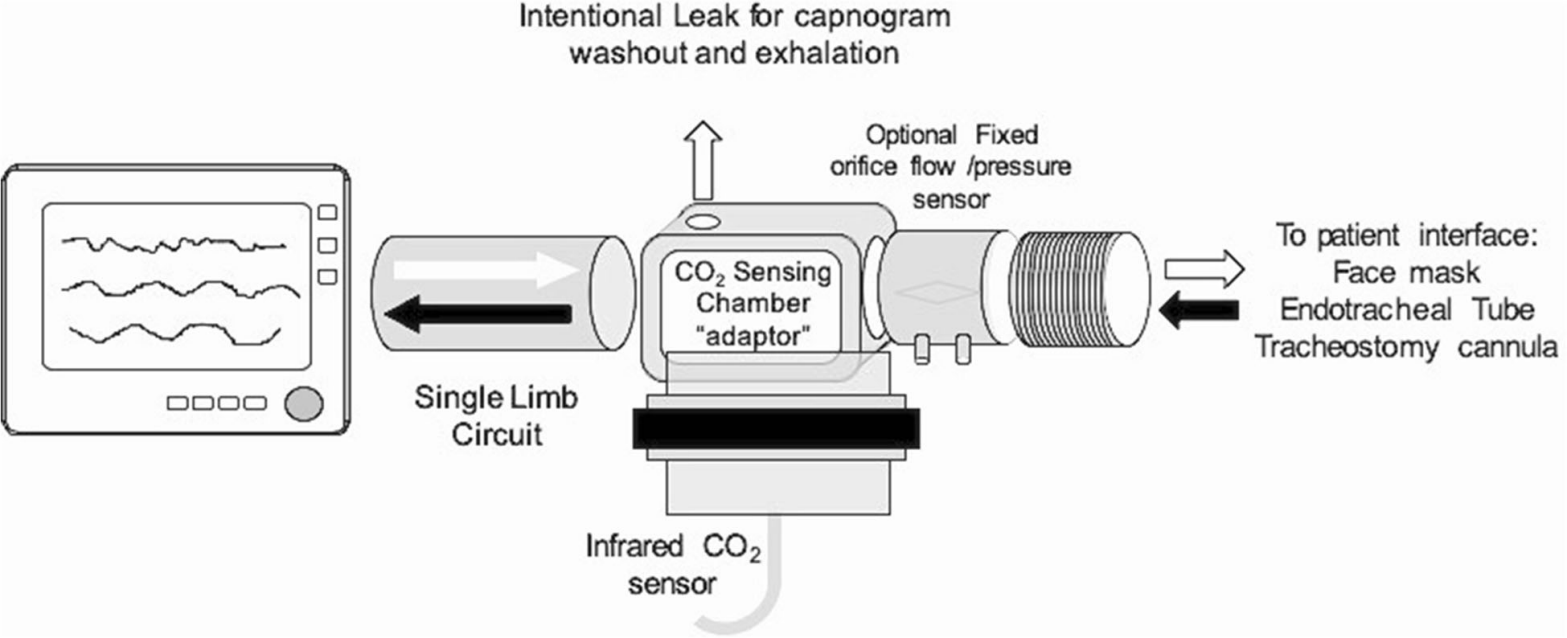
Experimental setup: single-limb circuit configuration. The experimental setup used to validate the end-tidal dilution method (etCO_2_D) to estimate auto-PEEP. See text for detailed explanations of the components. In this configuration, the exhaled air (black arrow) is always confronted with the continuous flow/pressure of fresh gas generated by the ventilator (white arrow). Whenever the end-expiratory pressure of the animal exceeds the pressure/flow coming from the ventilator, a complete normal capnogram is obtained from the CO_2_ sensor. If, however, the expiratory pressure of the ventilator exceeds the end-expiratory pressure of the patient, the capnogram will be diluted. In this evaluation, different levels of intrinsic PEEP were created and detected by stepwise increasing the level of end-expiratory pressure from the baseline value of 2 cmH_2_O in 1 and 0.2 cmH_2_O steps until de first dilution of the end-tidal portion of the capnogram was diluted. This pressure corresponded to the level of PEEPi

The principle is based on the analysis of the expiratory CO_2_ waveform, i.e., the capnogram either in the time or the volume domain. First, a conventional infrared CO_2_ sensor attached to its sensing chamber (commonly referred to as an adaptor) is placed as close as possible to the airway opening. This is between the face mask or the endotracheal tube and the distal end of the circuit in a single-limb circuit configuration or the Y-piece in a double-limb circuit configuration. Second, a small intentional leak must be included in the adaptor in order to allow for the dilution of the capnogram to occur. Third, in such setup, the exhaled air is always confronting a pressure or flow coming from the ventilator. Whenever the end-expiratory pressure from the lung is higher than the end-expiratory pressure coming from the ventilator, the exhaled breath will produce a normal, complete capnogram. However, whenever the expiratory pressure coming from the ventilator is higher than that from the patient or animal, the capnogram will be diluted by the fresh gas coming from the ventilator. The presence of the leak in the adaptor is therefore necessary to detect the CO_2_ dilution point during expiration, which corresponds to an equilibrium of expiratory pressures coming from the lung and the ventilator. Fourth, in the presence of PEEPi, by stepwise increasing the end-expiratory pressure of the ventilator, the first point at which the end portion of the capnogram is diluted will correspond to this equilibrium thereby providing a quantitative estimate of the level of PEEPi (Fig. 1). In the current evaluation, we have used a single-limb configuration and a non-invasive mechanical ventilator. The adaptor was a conventional CO_2_-Flow sensor (Respironics/Philips, Wallingford, CT, USA) used for volumetric capnography in which a simple small hole (2 mm) was drilled in the sensing chamber to create the intentional leak. As mentioned, the method also works in a double-limb circuit configuration in which the location in the center of mass of the two limbs is essential (Additional file 1: Figure S2 in the supplemental material for the double-limb configuration setup).

### Study protocol

To create different PEEPi conditions, an increased airway resistance was generated by the interposition of a fixed simple short 2–3-cm cylindrical tube with a reduced internal diameter (4.67 mm, 14F) at the airway opening before the flow sensor and the CO_2_ sensing chamber with the leak. The same resistance, CO_2_ sensor, and adapter (CO_2_-Flow sensor, Respironics/Philips Wallingford, CT, USA) were used in all experiments. All measurements were performed by the same researcher, always with the presence of at least one of the other research team members.

The different PEEPi conditions were then established by creating a combination of I:E ratios (by modifying inspiratory time and respiratory rate) and tidal volumes (by modifying inspiratory pressures). We used different inspiratory times from 30 to 80% (usually in 10% incremental steps), a respiratory rate from 20 to 30 bpm (in 5 bpm steps), and inspiratory pressures from 20 to 25 cmH_2_O, resulting in tidal volumes of 150 to 400 mL. To create the increasing levels of PEEPi, we used the inspiratory times as the reference combining the increasing values with different rates and inspiratory pressures. A minimum level of end-expiratory pressure of 2 cmH_2_O was maintained during all measurements. PEEPi levels were not randomized, and therefore, we introduced washout periods between each level to make conditions as independent as possible from each other. Washout periods consisted of reconnection to a Servo-i ventilator followed by 5–10 min of conventional ventilation using PEEP 2 cmH_2_O, respiratory rate of 20, I:E 1:2, and a FIO_2_ of 0.4. As the animals were healthy without parenchymal or airway disease, we considered this time enough for the lung to recover its baseline end-expiratory lung volume and alveolar end-expiratory pressure. This time has also been used in previous studies analyzing the respiratory system mechanics in pigs [8].

Airway pressure, flow, and exhaled CO_2_ were measured at the airway opening using a conventional fixed-orifice flow/pressure sensor (Respironics/Philips, Wallingford CT, USA) and recorded and analyzed by a customized software (DataCol, Respironics/Philips, Wallingford, CT, USA). At each experimental condition, the reference level of PEEPi was averaged from a triplicate occlusion measurement. Occlusions were performed by clamping the ventilator limb proximal to the pneumotachograph at early expiration identified visually on the pressure time waveform tracing. Immediately thereafter, expiratory pressure was increased from its baseline value of 2 cmH_2_O, in 1 cmH_2_O steps until two steps after the first dilution of the end-tidal portion of the capnogram was observed. Thereafter, the end-expiratory pressure was decreased in similar steps until the end-tidal dilution disappeared and a normal capnogram was restored. These two sequences were then repeated but applying PEEP steps of 0.2 cmH_2_O, the smallest change the experimental ventilator allowed. During the step PEEP changes, all other ventilator settings chosen to create the desired level of PEEPi (i.e., inspiratory time, respiratory rate, and inspiratory pressure) remained unchanged.

**Fig. 2:**
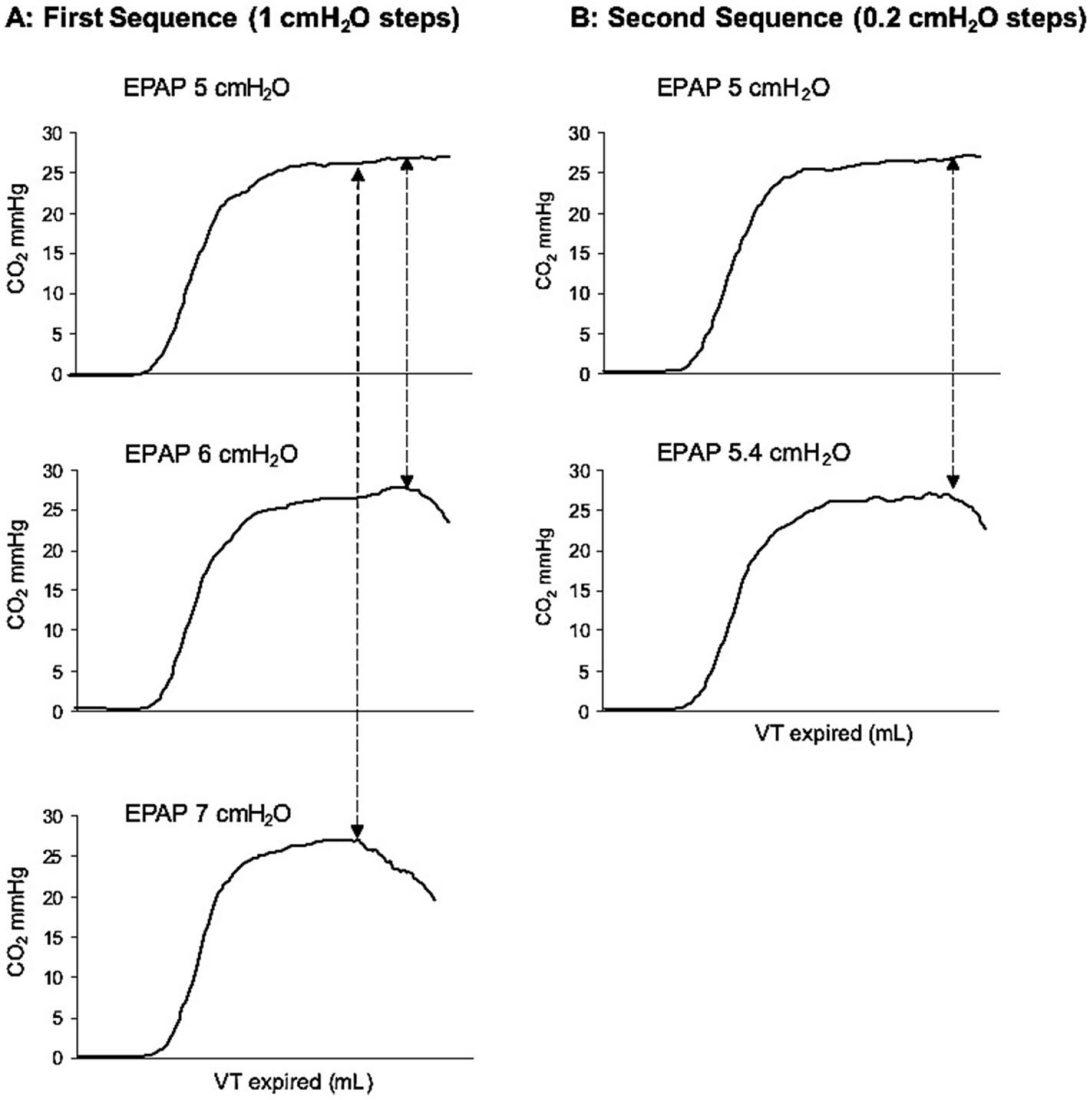
Representative volume-based capnograms during incremental PEEP with 1 cmH_2_O (**a**–**c**) and 0.2 cmH_2_O (**d**, **e**) steps to determine the etCO_2_D PEEPi. The previously determined occlusion method yielded a PEEP value of 5.4 cmH_2_O. **a** A full normal capnogram is represented. **b** When the expiratory pressure in the ventilator is increased from 5 to 6 cmH2O, the end-tidal dilution becomes visually evident. **c** When the end-expiratory pressure is increased further to 7 cmH_2_O, the capnogram becomes diluted at an earlier point during expiration

The expiratory pressure level corresponding to the first detected dilution of the end-tidal portion of the capnogram (etCO_2_D) was identified and compared with the level obtained by the occlusion method (OM). Figure 2 illustrates an example of PEEPi estimation based on the etCO_2_D method.

## Statistical analysis

Data are expressed as mean ± standard deviation unless specified otherwise. Normality was assessed by the Kolmogorov-Smirnov test. Correlation between PEEPi measured by OM and etCO2D was expressed as the squared Pearson’s coefficient. Difference between these two measurements was tested by Student’s *t* test, and Bland-Altman analysis was used to compare the agreement between them. Statistical analysis was performed using the SPSS software (SPSS 19.0; IBM, Chicago, IL). Measurements were treated as independent. In order to control the interventions by the effect of using several measurements in the same pig, we tested our results post oc by means of a mixed effect linear model using animals as a random effect. An intraclass correlation coefficient was calculated to describe to what extent the dilution method resembles the occlusion method accounting for the random effect that could have been introduced by the animals. A *P* ≤ 0.05 was considered as statistically significant. An estimation of the sample size was not made since we did not know the magnitude of the effect (the difference between the measurements), and therefore, the number of measurements in this exploratory study was decided arbitrarily. A sample size of 50 measurements was used in the current analysis.

## Results

Twelve female largo white, 4–6-week-old pigs (median weight, 25 kg [minimum to maximum, 23 to 27 kg]) were included in this analysis. A total of 52 PEEPi levels in 12 animals were analyzed. Those levels were achieved by inspiratory times of 1.4 ± 0.5 s (range 0.6–2.4), respiratory rates of 24.6 ± 3.4 bpm (range 20–30), and inspiratory pressures of 22 ± 2.2 (range 20–25 cmH_2_O). Figure 2 illustrates an example of how the etCO_2_D detected the level of PEEPi. In Table 1, we present our data rearranged and grouped according to different levels of PEEPi: low, moderate, and high with the range of ventilator settings used.

**Table 1:** Arrangement of data according to low, moderate, and high levels of PEEP

PEEP level	Ventilator settings			OM (cmH_2_O)	etCO_2_D (cmH_2_O)
	TI (%)	RR (bpm)	Insp. Pressure (cmH_2_O)		
Low	30–40	20–25	20–25	3.01 ± 0.73	3.66 ± 0.93
Moderate	40–60	25–30	20–25	5.49 ± 1.38	5.19 ± 1.05
High	50–80	25–30	20–25	8.97 ± 1.51	7.87 ± 1.42

*TI* inspiratory time, *RR* respiratory rate, *Insp. Pressure* inspiratory pressure, *OM* occlusion method, *etCO*_*2*_*D* capnogram dilution method

**Fig. 3:**
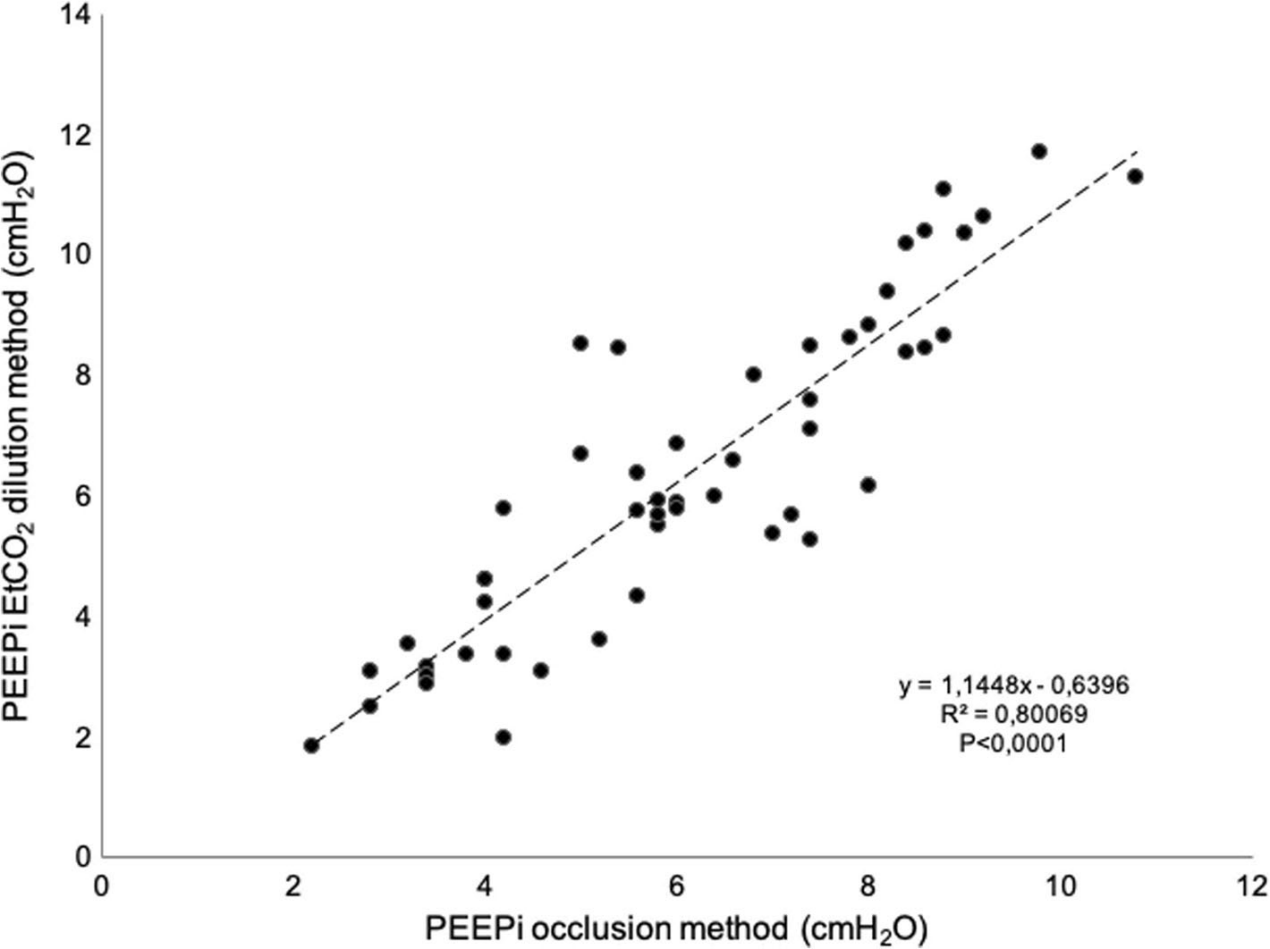
Regression analysis. Reference PEEPi (OM) and etCO_2_D PEEPi method had a good linear correlation (*R*^2^ 0.80, *P* < 0,001)

The etCO_2_D method detected step changes of 0.2 cmH_2_O as confirmed by the good correlation obtained between both methods (*R*^2^ 0.80, *P* < 0.001), Fig. 3. No differences were found between OM PEEPi 6.19 ± 2.11 cmH_2_O and EtCO_2_D PEEPi 6.45 ± 2.70 cmH_2_O; *P* < 0.142.

**Fig. 4:**
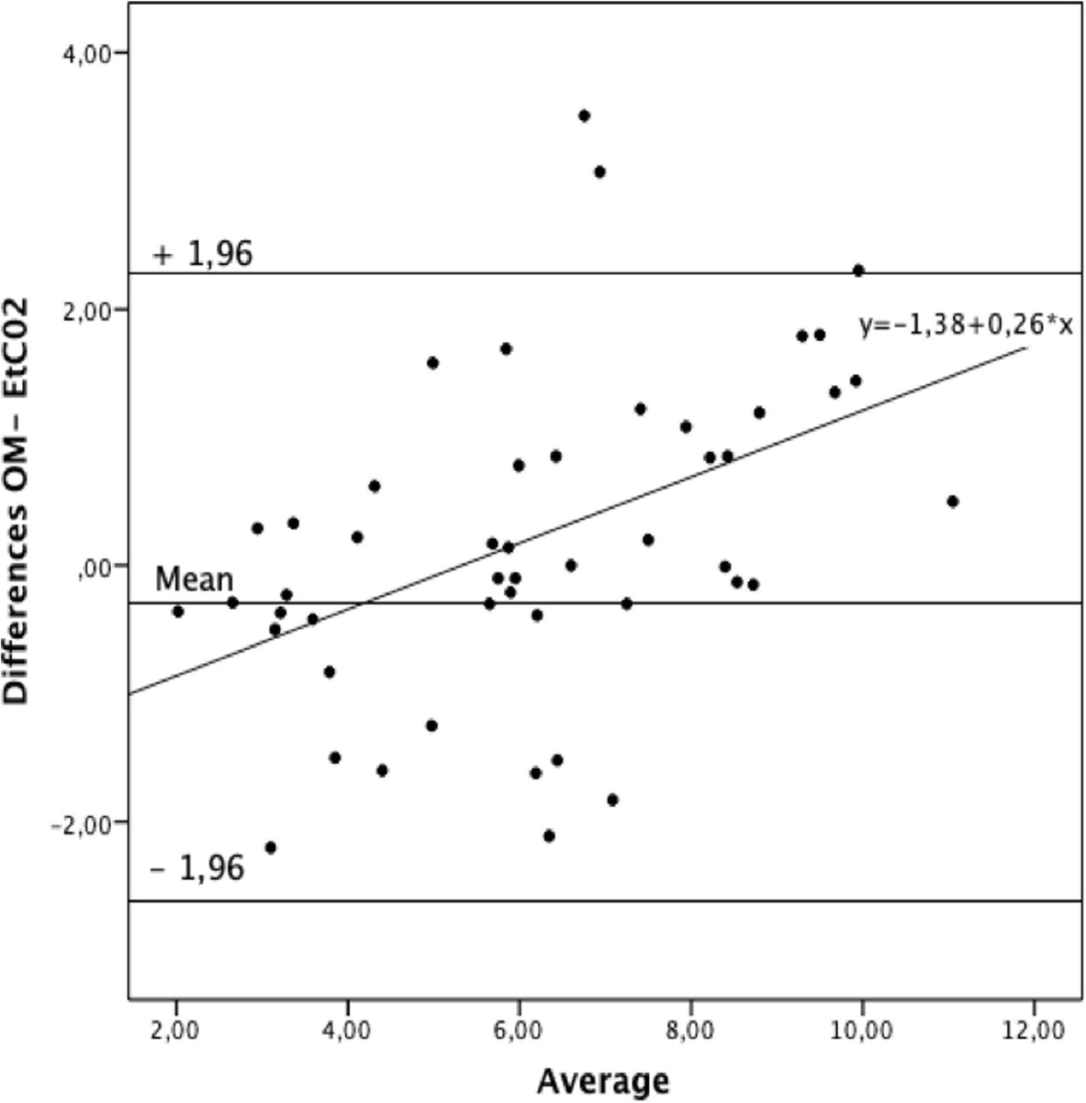
Bland-Altman analysis between the two PEEPi methods, OM minus etCO2D. OM, occlusion method

The reference and the etCO_2_D methods also presented a good agreement, bias − 0.26, and limits of agreement ± 1.96 SD (2.23; − 2.74 cmH_2_O; Fig. 4). A significant trend was observed in the Bland-Altman plot resulting in a regression equation of *Y* = − 1.38 + 0.26*Χ*; *R*^2^ 0.237 Sig < 0.0001. This suggests a possible underestimation by the etCO_2_D method at higher PEEPi levels.

Although we considered “a priory” that measurements were independent as they were made under different ventilatory conditions after a washout period and that most variability would come from the artificially created PEEPi, we tested whether our assumption was correct. For this purpose, a mixed effect linear model using animals as a random effect was performed. The likelihood ratio test comparing such a mixed effect model against a one-level ordinary linear regression was not significant (*P* = 1). Also, the slope of the relationship was the same in the two tested models with only a small difference in confidence intervals (0.69 [0.61–0.79] for a mixed effect model vs 0.69 [0.60–0.79] for an ordinary linear regression. Finally, the Akaike information criteria were similar for both models (148 for the mixed effect models vs 144 for the ordinary linear regression). Intraclass correlation coefficient for the overall data was 0.87 (95% confidence interval 0.78; 0.92).

Incremental and decremental end-expiratory pressure levels in both 1 and 0.2 cmH_2_O PEEP steps always resulted in exactly the same PEEPi values. We therefore assume that no major changes in lung heterogeneity due to atelectasis influenced our measurements.

## Discussion

In this study, we are presenting the first validation of a novel non-invasive method based on expired CO_2_ kinetics measured by conventional capnography for measuring PEEPi under passive ventilatory conditions. We compared its performance with the clinical reference occlusion method. We found good accuracy and acceptable precision for this new method making it a potentially helpful monitoring option to detect PEEPi during mechanical ventilation. The potential advantage of this method is that it can estimate PEEPi in non-passive breathing conditions and could therefore be useful to estimate PEEPi during spontaneous breathing during invasive mechanical ventilation. As PEEPi changes dynamically, the system could be useful to detect and monitor these changes by modifications in the end-tidal dilution pattern or by changes in the level of external PEEP or CPAP.

The presence of PEEPi can have important clinical consequences such as lung hyperinflation, barotrauma, increased work of breathing, patient-ventilator asynchrony, weaning failure, and impaired hemodynamics with decreased blood pressure and cardiac output.

A recognized problem is the difficulty to clinically measure PEEPi especially in non-passive conditions. The reference method for the clinical estimation of PEEPi in static conditions is the end-expiratory occlusion method. Pausing expiration by performing an expiratory hold maneuver for 3–5 s allows alveolar pressure to equilibrate with the pressure sensor in the ventilator. Static PEEPi (PEEPi-stat), as measured by the occlusion method, represents the average end-expiratory elastic recoil of the respiratory system at the lung volume at which the airway occlusion occurs. During the airway occlusion, both stress adaptation phenomena and equilibration of lung units with different regional time constants after emptying occur. In addition, it will underestimate PEEPi when peripheral units occlude and lose communication with the central airways. In contrast, there is also the possibility to estimate a dynamic PEEPi (PEEPi-dyn) which reflects the difference between the set external PEEP and the airway end-expiratory pressure at the onset of inspiratory flow. This can be easily done qualitatively by simple observation of the pressure-time and flow-time ventilator waveforms.

If the physiological meaning of PEEPi-stat is rather straightforward, that of PEEPi-dyn is still under debate [9]. It is said to represent the lowest regional pressure coming from early-emptying alveoli, i.e., those with the shortest time constants [10–12]. It is thus the lowest initial pressure that must be overcome either by the muscular effort or by the positive pressure of the ventilator to start the inspiratory flow. In normal healthy lungs, both static and dynamic PEEPi should have very similar values. However, in heterogeneously diseased lungs such as in severe obstructive pulmonary disease, PEEPi-stat is usually much higher than PEEPi-dyn reflecting the wide range of time constants and the dissipation of lung viscoelastic forces. This systematic underestimation of PEEPi-stat (measured by the occlusion method) by PEEPi-dyn has been well described during spontaneous breathing [13] as well as during assisted [14, 15] and controlled [16] mechanical ventilation. The difference can be quite large during controlled mechanical ventilation with reported PEEPi-dyn values of only 25–30% of measured PEEPi-stat [17]. We believe that the proposed etCO_2_D method measures a PEEPi-dyn as it detects the dilution in non-static conditions which should reflect the lowest PEEPi value of the system corresponding to the most early emptying units. We chose a model of healthy, non-heterogeneous lungs for our first validation in order to obtain the most similar values between the occlusion and the dilution methods.

When rearranging our data in three groups according to high, moderate, and low PEEPi values (Table 1), we found that at lower and moderate PEEPi values, the dilution method had the lowest differences with the occlusion method (average of the differences of − 0.64 cmH_2_O ± 0.19). At the highest levels of PEEPi however, we always found that the dilution method resulted in lower values than the occlusion method with an increased average of the differences of 1.10 ± 0.08 cmH_2_O. Nevertheless, at the highest air trapping conditions, the difference between the methods was less than 1.88 cmH_2_O. This confirms that we studied fairly homogeneous lungs and that these differences may also be related to gravitational changes in normal lungs. This could explain the trend observed in the Bland-Altman plot where at higher levels of PEEPi-stat, the etCO_2_D PEEPi progressively underestimated PEEPi-stat.

There are other several newly described methods to measure PEEPi. The latest generation ventilators continuously measure instantaneous airway pressure and flow with high accuracy. By introducing short expiratory holds, PEEPi can be continuously or semi-continuously measured using multiple linear regression and least-squares fit analysis [3]. Bellani et al. [18] recently described a method to estimate auto-PEEP based on the delay between the onset of the electrical activity of the diaphragm and the inspiratory flow. However, this method requires a special nasogastric tube, the EAdi catheter, and can only be applied with one specific mechanical ventilator.

## Limitations

Our study has a number of limitations that need to be addressed. As mentioned, this is a limited first evaluation study performed in a rather small number of animals. Even though the steps were not randomized and an average of 4 to 5 measurements were performed in each animal, the introduction of washout periods allowed us to analyze 52 independent PEEPi measurements as the mixed effect linear model confirmed. Our model included only healthy animals, and there is currently no information on how the method performs in heterogeneous lung conditions. Furthermore, this first experimental evaluation PEEPi was artificially created and does not reflect true clinical conditions. Our results should therefore be interpreted with caution. As the method is based on the detection of a difference in expiratory pressure between the subject and the ventilator at the airway opening, it should be able to detect any difference also in patients; the exact meaning of which and its real clinical value and use will have to be elucidated in studies involving patients with PEEPi related to pathological conditions of the lung. As discussed earlier, the proposed etCO_2_D method measures PEEPi-dyn, and therefore, differences with the occlusion method are expected to be larger in heterogeneous lungs. This could however be an advantage when monitoring during mechanical ventilation as the PEEPi-dyn is the one acting during active breathing and has important clinical consequences as it reflects the pressure needed to be overcome to start inspiration. However, any translation of our findings or interpretations to human injured lungs cannot be made at this stage.

Finally, we have evaluated this method only during passive breathing. According to its working principle, it should also be able to provide an equally good estimate in spontaneously breathing conditions but this will have to be validated in future studies.

## Conclusions

In this experimental model of healthy homogeneous lungs, under passive breathing conditions, the presented end-tidal dilution method provided good estimates of the level of intrinsic PEEP when compared with the clinical reference occlusion method.

## Additional files

Additional file 1: **Figure S1.** Experimental setup in a single-limb circuit configuration. **Figure S2.** Setup for a double-limb circuit configuration. (ZIP 161 kb)
