# Supplementary material for: Continuous monitoring of intrinsic PEEP based on expired CO2 kinetics: an experimental validation study
Source: Crit Care. 2019 May 29;23:192. doi: 10.1186/s13054-019-2430-9 (PMC6540388; doi:10.1186/s13054-019-2430-9)
Supplement: Supplementary file 1 — Figure S1. Experimental setup in a single-limb circuit configuration. Figure S2. Setup for a double-limb circuit configuration. (ZIP 161 kb) [file 13054_2019_2430_MOESM1_ESM.zip › R1. On line supplemental material.docx]

**On line supplemental material**

**Continuous Monitoring of intrinsic PEEP based on expired CO_2_ kinetics. An experimental validation Study.**

**Sarah Heili-Frades^1,3^, Fernando Suarez-Sipmann^2,3,4^, Arnoldo Santos^5^, Maria Pilar Carballosa^1^, Alba Naya-Prieto^1^, Carlos Castilla-Reparaz^6^. Maria Jesús Rodriguez-Nieto^1,3^, Nicolás González-Mangado^1,3^, German Peces -Barba^1,3^**

^1^ Intermediate Respiratory Care Unit. Pulmonology Department. IIS-Fundación Jiménez Díaz. UAM. CIBERES.

^2^ Servicio de Medicina Intensiva, Hospital Universitario de la Princesa. Madrid , Spain.

^3^ CIBER de enfermedades respiratorias. Instituto Carlos III, Madrid, Spain

^4^ Department of surgical Sciences, Section of Anesthesia and Critical Care, Uppsala

University Hospital, Hedenstierna Laboratory, Uppsala, Sweden

^5^ ITC Ingeniería y técnicas clínicas. CIBER de enfermedades respiratorias (CIBERES).

^6^ IIS-Fundación Jiménez Díaz. Department of Experimental Surgery.

**Figure 1**: Experimental set up in a single limb circuit configuration.


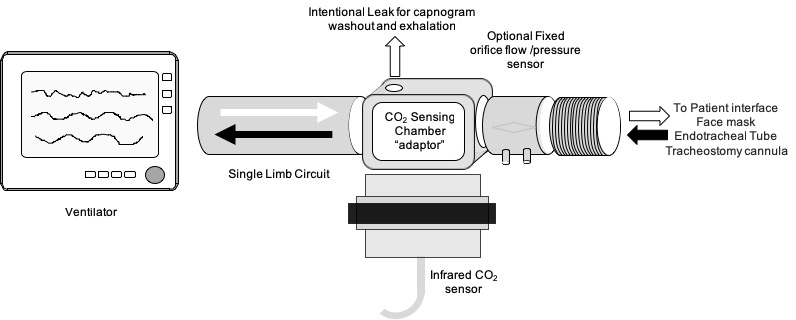


This figure depicts the experimental set-up used to validate the end-tidal dilution method (etCO_2_D) to estimate auto PEEP.

The ventilator was a non-invasive single limb BiPAP respirator (Respironics BiPAP ST).

The conventional CO_2_/Flow adaptor (Respironics/Philips, Wallingford, CT, USA) used included the CO_2_ sensing chamber and a fixed orifice flow/pressure sensor. A conventional clinically used infrared CO_2_ sensor included in the NICO monitor (Respironics/Philips, Wallingford, CT, USA) was used. A small intentional leak was created by drilling a 2 – 3 mm hole in the sensing chamber.

In this configuration the exhaled air (black arrow) is always confronted with the continuous flow/pressure of fresh gas generated by the ventilator (white arrow). Whenever the end-expiratory pressure of the animal exceeds the pressure/flow coming from the ventilator a complete normal capnogram is obtained from the CO_2_ sensor. If however the expiratory pressure of the ventilator exceeds the end-expiratory pressure of the patient the capnogram will be diluted. In this evaluation different levels of intrinsic PEEP were created and detected by stepwise increasing the level of end-expiratory pressure form the baseline value of 2 cmH2O in 1 and 0.2 cmH2O steps until de first dilution of the end-tidal portion of the capnogram was diluted. This pressure corresponded to the level of PEEPi.

The method can also be used in a double limb circuit configuration as used by conventional mechanical ventilators.

**Figure 2:** Set up for a double limb circuit configuration:


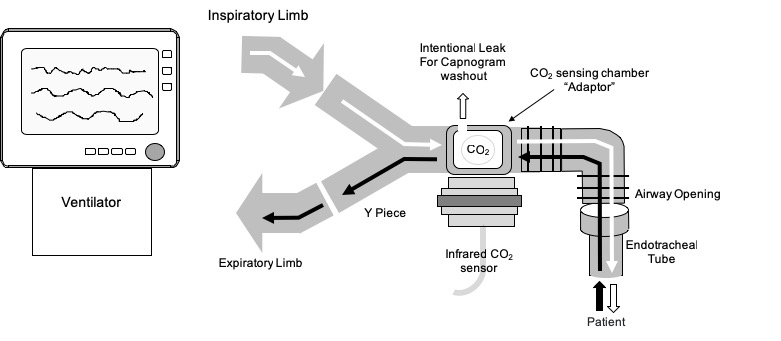


In this configuration the sensing chamber must be placed after the Y piece as close as possible to the airway opening. The operation principle is the same. To be fully operative in a double limb mechanical ventilator, the intentional leak to create the dilution should be quantified and accounted for by the ventilator if a continuous monitoring is to be applied.
