# Supplementary figures and images for: Continuous monitoring of intrinsic PEEP based on expired CO2 kinetics: an experimental validation study
Source: Crit Care. 2019 May 29;23:192. doi: 10.1186/s13054-019-2430-9 (PMC6540388; doi:10.1186/s13054-019-2430-9)

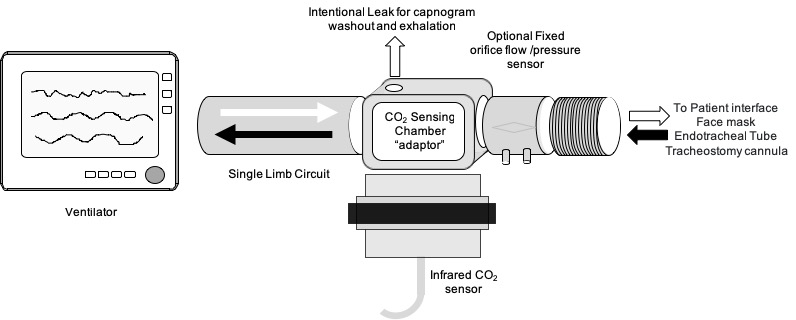

Supplement: Supplementary file 1 — Figure S1. Experimental setup in a single-limb circuit configuration. Figure S2. Setup for a double-limb circuit configuration. (ZIP 161 kb) [file 13054_2019_2430_MOESM1_ESM.zip › Figure 1 Supplemental material- R1.jpg]

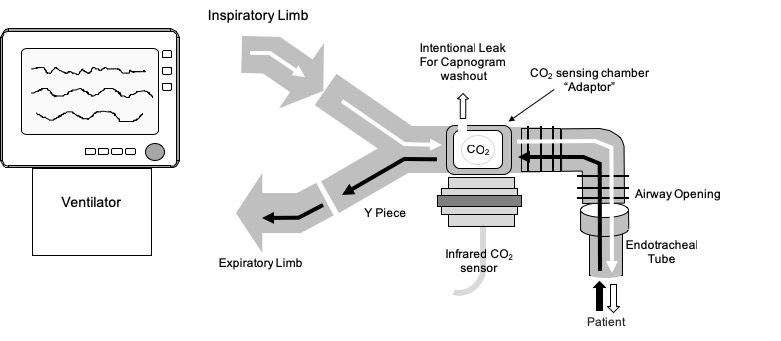

Supplement: Supplementary file 1 — Figure S1. Experimental setup in a single-limb circuit configuration. Figure S2. Setup for a double-limb circuit configuration. (ZIP 161 kb) [file 13054_2019_2430_MOESM1_ESM.zip › Figure 2 Supplemental material.jpg]
